# Translating tools for better parent-based assessment: An exploratory study

**DOI:** 10.4102/sajcd.v62i1.73

**Published:** 2015-06-06

**Authors:** Shabnam Abdoola

**Affiliations:** 1 Speech Language and Audiology, University of Witwatersrand, South Africa

## Abstract

**Background:**

Current speech language assessment and intervention measures are not always culturally valid, as they are not standardised specifically for the various cultural groups within the South African population; and thus need to be adapted.

**Objectives:**

The objective of this study was to examine the appropriateness and utility of translations of the Ages & Stages Questionnaire (ASQ) instrument (60 month age group) from English to the Hindi language and culture, which is represented in South Africa.

**Methods:**

Biographical questionnaires, ASQ and evaluation thereof were translated in Hindi and completed by parents of 15 typically developing South African preschool children of Indian origin, at the 60 month age level (including children between 57 and 66 months).

**Results:**

Participants reported that the questions were well phrased, and that illustrations and tips helped them to complete the questionnaires quickly and accurately. They preferred to be questioned in Hindi, which helped them understand the questions and made it easier to provide the necessary information to answer the questions.

**Conclusions:**

In conclusion, it is evident that this translation of the ASQ (60 month age group) from English to Hindi served as an appropriate tool for use with the middle socioeconomic class Hindi (Indian) language and culture. The results of this study would assist to determine the functionality of culturally and linguistically valid assessment tools for different populations, and would contribute to the development of Early Childhood Intervention as a whole in South Africa. It would also contribute to the development of multilingual informal school-readiness screening questionnaires appropriate for the South African context. This is particularly relevant, as school-readiness assessments take place at 60 months to ensure that the child is ready to learn by school age (6–7 years).

## Introduction

South Africa has a diversified population as well as a large influx of international immigrants. As a result, there are many people in South Africa that speak languages other than the eleven official ones as their first language. As stipulated by the Constitution of the Republic of South Africa (1996), national legislation requires the promotion, development, respect and use of all official languages, indigenous languages, sign language, and all languages commonly used by communities in South Africa (including German, Greek, Gujarati, Hindi, Portuguese, Tamil, Telegu and Urdu).

The South African population is rich in diversity regarding language, culture and religious beliefs. Levin (2006) highlighted cultural mismatch and language barriers as obstacles to efficient communication, as many therapists speak only English or Afrikaans. Although current assessment and intervention measures are available in English, these are not always culturally valid, as they are not standardised specifically for the South African population, and thus need to be adapted (Kanjee, 2005; Pena, 2007). Kanjee (2005) highlights the necessity of developing or adapting assessment measures in South African society so that tests may be answered by, and are appropriate for test-takers from all cultural and linguistic backgrounds. Furthermore, adapting and/or translating measures serves to enhance fairness in assessment (thereby minimising bias and increasing the validity of the responses), reduce costs, save time, as well as facilitate comparative studies between different cultural and language groups within a given context (Kanjee, 2005).

Various communication strategies are used, such as utilising the family's prime or home language or providing translation and bilingual services when communicating with parents and families. The use of nonverbal actions and paralinguistic features such as timing, tone and emphasis could encourage or hinder parent contributions, which may vary on a continuum within a given culture or between different cultures, and motivates the adaptation of measures to ensure cross-cultural validity and develop cultural competence of interventionists (Lynch & Hanson, 2004). Open communication contributes to the effectiveness of intervention, and allows for families to guide interventionists to what they see as most important as emphasised by the transactional model of service delivery (Winton, McCollum & Catlett, 2004). In light of the fact that it is estimated that more than 800 000 individuals, including Indian and Pakistani immigrants, in South Africa speak dialects of a common root language, Hindi, as a first language it is essential to have culturally appropriate assessment materials to measure the language and development of children from these cultures in South Africa. In South Africa, there are currently no developmental assessments or parent questionnaires in Hindi; indicating a paucity of developmental outcomes for the target culture in the South African population. Translating an assessment into Hindi provides a tool that is easier for Hindi-speaking parents to understand and respond to, thus providing a more accurate assessment of their child(ren); and ultimately aiding the process of establishing partnerships and, in accordance with Briggs (1998), building bridges with families of the different communities particularly within South Africa.

As a shift towards the importance of family inclusion is identified in researched service delivery approaches, it is of importance to note that collaboration with families is essential in Early Childhood Intervention (ECI) (Dunst, Bruder & Espe-Sherwindt, 2014; Guralnick, 2011). It is important to realise that the assessment process, which is likely to introduce parents to Early Childhood Intervention, may facilitate discussion as well as help to consolidate parents’ and professionals’ views, thus promoting that collaboration. This is particularly relevant in many communities within South Africa, where negative views of disability prevail. In doing so, we could establish trusting relationships with families with children at risk, and offer both support and education to them, thus becoming ‘brokers of information’, as advocated by Rossetti (2001). This facilitates the process of bringing ECI services, and the families and children who need them, together. There is a growing interest in parent-based assessment, as trends indicate that parental reports of their child's skills are often predictive of developmental delay; and parental concerns about language, fine motor, cognitive, and emotional and behavioural problems are highly predictive of true problems. By collaborating from the beginning, parents and professionals are better able to develop synthesised goals, and parents may be more likely to follow through on recommendations; resulting in more sustainable ECI services in South Africa.

Families have been viewed as developmental, functional environments in which integrated and continuous holistic assessment and intervention, across interrelated areas of development should be conducted (Bjorck-Å kesson, Carlhed & Granlund, 1998). Effective assessment and intervention with young children is based on the systems approach to understand the reciprocal interaction and stressors of child competencies, family patterns of interactions and family resources (Guralnick, 2011. By acknowledging and assessing these interactions, interventionists are better able to understand and improve on the cascading effects of influential changes within the interrelated systems of family, environment and community on those children and their families; and thus improve and optimise situations for supporting and enhancing development (Meisels, 2001; Shonkoff & Meisels, 2000).

The Ages and Stages Questionnaires (ASQ) (Squires, Potter & Bricker, 1997; Appendix A), a reliable parent/caregiver-completed developmental screening tool for children in their first five years, has the advantage of parents being active participants in the evaluation of their children, and although several successful and reliable cross-cultural adaptations have been made and it has been translated into Afrikaans (Bornman, Sevcik, Romski & Pae, 2010), there are no documented studies on the use of this questionnaire in the Indian (Hindi) context in South Africa. The ASQ allows professionals to quickly identify young children at risk for developmental delays, identify behaviours of concern to caregivers, and identify any need for further assessment (Bornman *et al.*, 2010; Squires *et al.*, 1997). It encompasses five developmental areas, with six questions in each of the following: communication, gross motor, fine motor, problem-solving, and personal social development. The ASQ is noted by its publishers as having 94% reliability and between 75% and 89% validity. It is written in simple language, and includes illustrations and numerals, and is thus functional in contexts where people may have limited literacy skills, such as in certain parts of South Africa (Bornman *et al.*, 2010; Levin, 2006; Salisbury, 1992). Thus, the objective of this study was to determine the appropriateness of translations of the ASQ instrument (60 month age group) from the English to Hindi language and culture. This age group was selected, as it would be the ideal age for informal school-readiness screening assessments. As there is no standard prescribed curriculum for pre-school, there are often inter-school discrepancies in the facilitation & mastery of school-readiness skills. It is only at the age of 5 years (60 months) that assessments are completed to ensure that the child is ready to learn by school age (6–7 years) (Davin & Van Staden, 2012).

## Research aim

The study aimed to examine the utility of linguistic translations of the ASQ instrument (60 month age group) from English to Hindi.

### Research methods

#### Ethical clearance

Ethical clearance was obtained from a South African university's Non-Medical Ethics Committee (protocol number: H14/04/35).

## Sample

Convenience sampling was used to recruit participants for this study, who were selected because they were accessible, could express themselves in Hindi, and had children between the ages of 57 and 66 months. The study was contextual and descriptive, and the researcher aimed for the sample to be representative of the target language and culture.

Written informed consent was obtained from the participants. The biographical information questionnaire, ASQ and an evaluation thereof were completed by parents of 15 South African preschool children of Indian origin, at the 60 month age level (including children between 57 and 66 months). Participants were identified within the same, predominantly Indian, community and cultural group, in the same geographical area (Laudium) of Gauteng, South Africa. Participants were of middle class socioeconomic status, thus implying that at least one parent in each family had completed a high school education and were formally employed. They typically lived in apartments or houses with basic amenities such as indoor plumbing and electricity, and owned at least one vehicle per family. The family structures included both-parent as well as single-parent households, and blended households (following divorce or death). The number of children in each household varied between one and four, with an average of two children per family. Children in these households were likely to have their own books and toys.

The mean age of the children was 61.8 months, ranging from 57‒66 months. The chronological age of the children was distributed as follows: 3 children at 66 months of age, 3 children at 63 months of age; 2 children at 62 months of age; 1 child at 61 months of age; 2 children at 60 months of age; 3 children at 59 months of age, and 1 child at 58 months of age. Three fathers and twelve mothers participated in the study. The parents’ first language and thus their children's mother tongue was Hindi. Two of the fathers of the six boys and nine girls were employed full-time, one was unemployed. Eight of the mothers of the six boys and nine girls were full-time home executives, two worked full-time, and two worked part-time.

## Inclusion and exclusion criteria

A requirement of the study was that parents had to be able to read Hindi (self-reported), in order for them to complete the questionnaire and the ASQ (Bornman *et al.*, 2010).

## Method

### Translation of the ASQ

Informed consent was obtained from all the translators; and a biographical information questionnaire, the selected ASQ age group questionnaire (including its instructions), as well as an evaluation guides for parents, were translated from English to Hindi by translators that were literate and fluent in both English and Hindi (at reading, speaking and writing levels). Separate translators, with experience of teaching Hindi and Hindi-English translation, were asked to evaluate and improve the quality of the translated documents before they were then back-translated; whereby a second set of translators translated the Hindi versions into English, and the two English versions were then compared to identify and resolve any differences (Kanjee, 2005; Pena, 2007). This served to ensure that linguistics in both the English and Hindi versions conveyed the same meaning and used the correct and relevant vocabulary, and thus enhanced the accuracy of the translation. Careful attention was given to the translation of grammatical constructs which could possibly contribute to meaning or provide cues which may influence the way target behaviours are elicited, and the same illustrations were used in the Hindi version as in the English version of the ASQ (Bornman *et al.*, 2010; Pena, 2007) thus ensuring that the English and Hindi versions conveyed the same meaning and allowed examination of the same constructs for each item (Pena, 2007).

**FIGURE 1:**
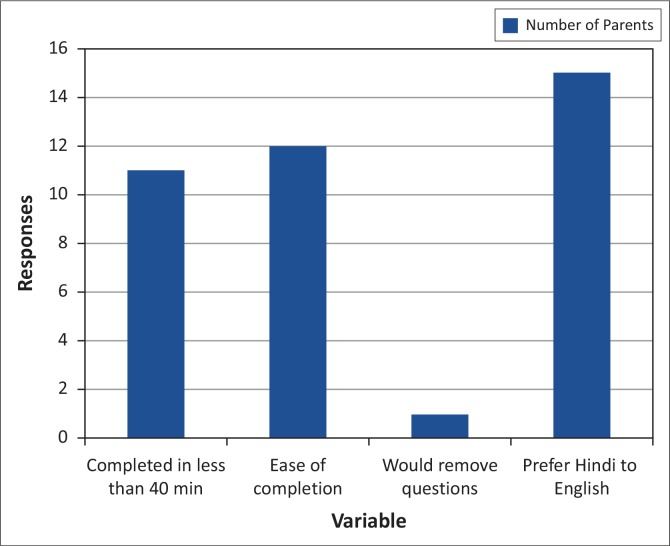
Parent responses.

### Testing the use of the translated version on Hindi-speaking parents

Instructions were provided to parents, thus preventing differences of outcomes related to differences in expectations or interpretation; and they were asked to complete and return the documents timeously (Pena, 2007). After completing the biographical questionnaire, parents were expected to complete each of the 30 developmental items on the Hindi ASQ questionnaire with their child. For each item, they were to indicate one of three responses: ‘Yes’, ‘Sometimes’, or ‘Not yet’; which are scored as 10, 5, or 0, respectively and added to obtain total domain scores. Scores could then be copied onto a simple grid that could give an at-a-glance picture of developmental skills. One or more scores in the grid's dark-shaded zone indicated that the child could need further assessment. Scores in the light shaded ‘monitoring’ zone could help identify children at risk, and allow for professionals to give parents activities to help their child make progress in these areas before their following screenings. Scores outside the shaded zones indicated that the child was developing appropriately in these areas. After completing the ASQ questionnaire, parents were asked to complete an evaluation thereof, which included questions regarding administration time, ease of using the ASQ questionnaire, as well as linguistic and cultural relevance of the questionnaire.

## Analysis of results

When asked to evaluate the Hindi ASQ, 11 out of 15 parents reported that it took them between 20–40 minutes to complete the questionnaire, and three reported that it took longer than 40 minutes. Twelve of the 15 parents found the questionnaire easy to complete, and reported that the questions were well phrased, and that illustrations and tips helped them to complete the questionnaires quickly and accurately. Three parents reported that, although they were able to answer the questions, they were somewhat unfamiliar with some concepts which made it difficult to answer some questions accurately. Twelve parents reported that they were uncomfortable if they noticed any items that their children had not mastered, as developmental delays, disorders and illness in general, are commonly concealed and are viewed in a negative light by most members of the community. The majority of parents reported that they would not remove these questions from the measure, as they retrospectively realised that these were essentially important issues that required attention to promote their children's development. All the parents reported that they preferred to be questioned in Hindi, as this improved their confidence, helped them understand the questions and made it easier to provide the necessary information to answer the questions. They were now able to understand the rationale for many questions, the use of which they may not have been able to justify if they were completing the test in English.

In translating the ASQ, this study introduces a tool for use in the Hindi (Indian) language and culture, thereby addressing the paucity of measures for this culture and language in South Africa. However, this translation was completed for research purposes only, and not for clinical use. Thus, the Hindi translation cannot be obtained for commercial use. The study did not include children from different socioeconomic backgrounds or in younger age groups where timely intervention is likely to be most beneficial. However, it can be easily converted into other Indian languages and used to motivate further research, and develop more valid and focused tools for use within the Indian culture.

This exploratory study involved only a small number of participants, thus limiting generalisation to the wider community and different age groups of children within the target culture, and necessitating further large-scale community-based studies across different population groups and environments. Parents could interpret their children's behaviour and development according to their cultural expectations, and their reporting of information could be influenced by their cultural perceptions, assumptions and biases (Pena, 2007). A way to overcome this possible cultural bias and inappropriateness would be to conduct a study of item salience, as described by Pena (2007), which would also act as a comparative measure of cultural differences in future research. Further research could also address specific cultural factors such as the South African Hindi community's views on the causes of disability and their attitudes toward intervention. This could provide important information for early interventionists in South Africa. Hindi has many dialects and usages across and within itself. This serves as a major limitation, for which Pena (2007) suggests the development of parallel vocabulary measures, which would also improve psychometric equivalence.

Additional modifications may be necessary when translating this measure into different languages and for other cultures, as well as for adapting it for different socioeconomic groups and environments (Bornman *et al.*, 2010). This study may serve as a foundation and guideline for such adaptations, as it indicates some of the factors that are to be considered in cross-cultural adaptation (Bornman *et al.*, 2010; Kanjee, 2005; Pena, 2007). It also serves to overcome obstacles such as language barriers, which may limit the success of early intervention (Levin, 2006).

In conclusion, it is evident that this translation of the ASQ (60 month age group) from English to Hindi served as an appropriate tool for use with the middle socioeconomic class Hindi (Indian) language and culture. The research process was successfully implemented and the aims of the study were thus reached. The results of this preliminary study will assist to determine the functionality of culturally and linguistically appropriate assessment tools for different populations, and will contribute to the development of intervention as a whole and the improvement of the broader service delivery outcomes within vulnerable, multilingual and multicultural communities particularly in South Africa.

## Appendix A

**The Ages & Stages Questionnaire (ASQ) instrument (60 months age group) Hindi**
